# New-onset Postural Orthostatic Tachycardia Syndrome Following Coronavirus Disease 2019 Infection

**DOI:** 10.19102/icrm.2020.111102

**Published:** 2020-11-15

**Authors:** Khalil Kanjwal, Sameer Jamal, Asim Kichloo, Blair P. Grubb

**Affiliations:** ^1^McLaren Greater Lansing Hospital, Lansing, MI, USA; ^2^Hackensack University Medical Center, Hackensack, NJ, USA; ^3^Central Michigan University, Saginaw, MI, USA; ^4^The University of Toledo Medical Center, Toledo, OH, USA

**Keywords:** COVID-19, dysautonomia, orthostatic intolerance, postural orthostatic tachycardia syndrome

## Abstract

Coronavirus disease 2019 (COVID-19) is caused by severe acute respiratory syndrome coronavirus 2. We report a case of new-onset postural orthostatic tachycardia syndrome in an otherwise healthy female patient following COVID-19 infection. The patient presented with fatigue, orthostatic palpitations, dizziness, and presyncope. She underwent head-up tilt-table testing and the findings were suggestive of postural orthostatic tachycardia syndrome.

## Introduction

Coronavirus disease 2019 (COVID-19) is caused by severe acute respiratory syndrome coronavirus 2 (SARS-CoV-2).^1^ First reported in Wuhan, China in late 2019, the virus has spread worldwide and triggered a global pandemic.^1^ As of early August 2020, almost 4.9 million patients have been diagnosed with COVID-19 in the United States and approximately 160,000 reported deaths attributed to the condition have occurred nationwide.

Although COVID-19 primarily affects the lungs, patients may have multiorgan system involvement with serious clinical outcomes.^2–4^ We report a case of postural orthostatic tachycardia syndrome (POTS) in an otherwise healthy female after COVID-19 infection.

POTS is a heterogeneous group of disorders resulting in a similar physiological state of orthostatic tachycardia and symptoms of fatigue, headache, dizziness, visual disturbances, and presyncope.^5–7^

Although the mechanisms for the development of POTS are not well-understood, patients with POTS often report experiencing a trigger such as a viral illness, electrical injury, lightning injury, gastric bypass surgery, or traumatic brain injury weeks to months prior to the onset of symptoms.^5–7^

## Case presentation

A 36-year-old otherwise healthy female presented with fever, fatigue, and shortness of breath. Given the global pandemic of COVID-19, she underwent SARS-CoV-2 viral serology testing, which yielded a positive result. She showed minimal symptoms and normal oxygen saturation as measured by a pulse oximeter. She was asked to self-quarantine and manage her symptoms with antipyretics and to report to the emergency room if her symptoms worsened. Ultimately, she felt better within a few days. Three to four weeks after being diagnosed with COVID-19, however, she began experiencing fatigue, headache, dizziness, chest pain, and palpitations, especially while getting up from the sitting position. She followed up with her primary care physician, who repeated the SARS-CoV-2 serology assessment, which yielded a negative result. As she was experiencing orthostatic palpitations and chest pain, she underwent electrocardiography, which showed sinus rhythm and a heart rate of 84 bpm, with no ischemic changes. A follow-up stress test and transthoracic echocardiography showed normal results. She also underwent brain magnetic resonance imaging for her dizziness and the result was normal as well. Her event monitor only showed episodes of sinus tachycardia.

Further examination in the physician’s office revealed a sitting heart rate of 86 bpm and blood pressure of 115/65 mmHg. Upon standing, however, her heart rate was 115 bpm and her blood pressure was 105/70 mmHg. She had normal cardiovascular, respiratory, gastrointestinal, and neurological examination results. Because of her orthostatic increase in heart rate, she underwent head-up tilt-table (HUTT) testing. The findings of her HUTT examination are shown in **Table 1** and were suggestive of POTS. She did not need isoproterenol or sublingual nitrate as she met criteria for POTS within nine minutes of upright tilt. Her clinical symptoms and the HUTT findings were consistent with the diagnosis of POTS. We suggested increased salt and water intake. In addition, the patient was given oral ivabradine 5 mg twice a day, which resulted in both subjective symptomatic improvement in orthostatic symptoms and tachycardia as documented during one of her follow-up visits, with a maximum increase in heart rate from 78 bpm to 90 bpm.

## Discussion

COVID-19 infection primarily affects the lungs; however, there is increasing evidence that it can involve multiple organs including from the cardiovascular, neurological, and renal systems.^1–4,8,9^ Some case reports of autonomic dysfunction presenting as sinus tachycardia, episodic sinus bradycardia, and sinus pauses in patients with COVID-19 infection have been published to date. Notably, these manifestations are usually seen in acutely sick COVID-19 patients.^10,11^

There are also reports of cardiac involvement that persist in the recovery phase as well. In a recent study of cardiac magnetic resonance imaging in recovered COVID-19 patients, almost 78% had cardiac involvement and 60% had ongoing myocardial inflammation independent of the pre-existing condition.^12^

POTS usually affects young women of childbearing age and presents with symptoms of orthostatic tachycardia or palpitations; dizziness; chest pain; headache; fatigue; visual disturbances; and, rarely, presyncope and syncope. Usually, patients will report the occurrence of a triggering event weeks to months before the onset of POTS symptoms.^5–7^ These triggering events may include a viral illness, trauma, pregnancy, electrical or lighting injury, or bariatric surgery. There are reports suggesting that POTS may be an autoimmune disorder.^13–15^ In one study, POTS patients had elevated levels of autoantibodies that cross-reacted with beta and muscarinic receptors and resulted in tachycardia.^15^ POTS is usually diagnosed through a good history evaluation, physical examination, and review of the heart rate and blood pressure in sitting and standing positions. HUTT may help in difficult clinical scenarios but is mostly used to confirm the clinical diagnosis. A protocol used for tilt-table testing includes a 70° baseline upright tilt for a period of 20 minutes to 30 minutes with continuous heart-rate and blood-pressure monitoring. At our institution, we apply sublingual nitroglycerine 0.4 mg in the provocative phase of the tilt-table test if required. Patients are diagnosed with POTS if they have symptoms of orthostatic intolerance accompanied by a reproducible heart rate increase of at least 30 bpm or an absolute heart rate of more than 120 bpm that occurs in the first 10 minutes of assuming an upright posture or while on a tilt table.^5–7^

Our patient recovered from an acute COVID-19 infection and, three weeks to four weeks later, she began experiencing symptoms of fatigue, orthostatic palpitations, chest pain, lightheadedness, headache, and presyncope. The exact mechanism of post–COVID-19 POTS is unknown and, at best, speculative at this time. Given the lag period of a few weeks between the COVID-19 infection and the onset of POTS symptoms, autoimmunity is a likely mechanism. However, our patient was not tested for autoantibodies cross-reacting with other receptors such as beta or muscarinic receptors. It is possible that anti–SARS-CoV-2 antibodies may cross-react with the receptors in the ganglia, as has been proposed in earlier studies considering the autoimmune basis for POTS.^13–17^ Unfortunately, the patient was not tested for anti–SARS-CoV-2 antibodies.

Treatment protocols for POTS have been described in detail previously.^5–7^ Existing protocols include increased intake rates of dietary fluids and salt, physical counter-maneuvers, and aerobic/resistance training. If these therapies are ineffective, pharmacotherapy options including fludrocortisone, midodrine, pyridostigmine, and ivabradine are trialed. It is not uncommon for these patients to need multiple medications to achieve the control of symptoms. In our patient, the dominant symptom was orthostatic palpitation and ivabradine, a selective sodium (funny current) inhibitor, was prescribed to be taken together with increased water and salt intake. On follow-up, she demonstrated improvement in orthostatic tachycardia but continued to experience fatigue and the occasional headache. Physicians need to be aware that POTS may be a late complication of COVID-19 and it is important to make a correct diagnosis so that these patients may receive appropriate treatment.

## Conclusion

Autonomic dysfunction presenting as POTS can be a delayed manifestation of COVID-19 infection. A high index of suspicion in post–COVID-19 patients who present with orthostatic symptoms may lead to proper diagnosis and treatment in such patients.

## Figures and Tables

**Table 1:** HUTT Test Findings

Time	Heart Rate	BP	Pulse Oximetry	Symptoms
0 min	88 bpm	120/65 mmHg	98%	None
5 min	110 bpm	115/70 mmHg	97%	Dizziness
8 min (tilt was terminated)	129 bpm	110/70 mmHg	99%	Dizziness, palpitations, and a feeling that she was going to pass out
